# Widespread presence of novel gammaherpesviruses in lagomorph species (*Oryctolagus cuniculus*, *Lepus* spp. and *Ochotona alpina*)

**DOI:** 10.1186/s12985-025-03000-5

**Published:** 2025-12-24

**Authors:** Maria Carolina Matos, Joana Abrantes, Ana M. Lopes

**Affiliations:** 1 Centro de Investigação em Biodiversidade e Recursos Genéticos, CIBIO, InBIO Laboratório Associado, Universidade do Porto, Campus de Vairão, Vairão, 4485-661 Portugal; 2 BIOPOLIS Program in Genomics, Biodiversity and Land Planning, CIBIO, Campus de Vairão, Vairão, 4485-661 Portugal; 3 Departamento de Biologia, Faculdade de Ciências, Universidade do Porto, Porto, 4099-002 Portugal; 4 UMIB-Unit for Multidisciplinary Research in Biomedicine, ICBAS-School of Medicine and Biomedical Sciences, University of Porto, Porto, Portugal; 5 Laboratory for Integrative and Translational Research in Population Health, ITR, Porto, Portugal

**Keywords:** Gammaherpesvirus, Wildlife, New virus, Lagomorphs

## Abstract

**Background:**

Gammaherpesviruses co-evolve with their hosts, resulting in species-specific associations and restricted host tropism. In lagomorphs, six herpesviruses (LeHV-1 to LeHV-6) have been identified, with LeHV-4 being associated with mortality in European rabbits (*Oryctolagus cuniculus*), while the others cause asymptomatic infections. LeHV-5 has been hypothesized to contribute to high morbidity and mortality in Iberian hares (*Lepus granatensis*) when in presence of concomitant infections such as myxomatosis. However, herpesvirus infections in wild and domestic lagomorphs remain poorly understood.

**Methods:**

Here, we conducted the first large-scale screening for herpesviruses in lagomorphs. Using a generalist PCR, we analyzed over 1,000 DNA samples from European rabbits, hares (*Lepus* spp.), cottontails (*Sylvilagus* spp.), pikas (*Ochotona* spp.), pygmy rabbits (*Brachylagus idahoensis*), volcano rabbits (*Romerolagus diazi*), Amami rabbits (*Pentalagus furnessi*), and riverine rabbits (*Bunolagus monticularis*).

**Results:**

Herpesviruses were detected in 75 samples (7.24%), revealing a putative novel virus in pikas, with ~ 80% similarity to known gammaherpesviruses. We further show circulation of LeHV-5 in European and mountain hares for the first time.

**Conclusions:**

These findings expand the current knowledge of herpesvirus diversity in lagomorphs. Given their potential role in immunosuppression and disease interactions, particularly with myxoma virus, further research is needed to assess their impact on host health and population dynamics.

**Supplementary Information:**

The online version contains supplementary material available at 10.1186/s12985-025-03000-5.

## Background

Herpesviruses are often described as an evolutionary success. Their widespread presence across nearly all vertebrates, and even in some invertebrates, ranks them among the most ubiquitous and successful viral groups known. After acute primary infection, latency is established, with life-long infections alternating between cycles of virus replication and dormant infection [1]. Reactivation is triggered by stress, co-infections, pregnancy, among others, and contributes to their ubiquity [2–4]. Although typically benign, herpesvirus infections can result in reproductive failure, neonatal loss, or mortality in immunocompromised and/or non-definitive hosts [5, 6]. Both innate and adaptive host immunity play a role in determining the extent of clinical signs during the lytic stage [7]. Nonetheless, virus shedding may or may not be associated with the presence of clinical signs [8], hence, host infectiousness is also associated with asymptomatic reactivation.

Herpesviruses are large (200–250 nm in diameter), enveloped, double-stranded DNA viruses, classified into three main families and encompassing more than 100 species. Virions have a distinct morphology and are organized in four layers: a DNA core surrounded by a T = 16 icosahedral capsid, a tegument with virus-encoded proteins and a final layer of a lipid envelope containing viral glycoproteins [9]. The family *Malacoherpesviridae* comprises viruses that infect molluscs, while *Alloherpesviridae* includes viruses infecting fish and amphibians. *Orthoherpesviridae* encompasses viruses that infect mammals and birds and is subdivided into three subfamilies — *Alpha-*, *Beta-* and *Gammaherpesvirinae* — based on host range, genetic organization and replication strategies [9]. In addition to these distinct biological properties, classification is further supported by phylogenetic analyses [10]. Gammaherpesviruses infect naïve B cells, macrophages and epithelial cells and, unlike alpha- and betaherperviruses, are oncogenic, being associated with lymphoproliferative diseases, lymphomas and other cancers [11, 12]. Members of this subfamily include Epstein-Barr virus and Kaposi sarcoma-associated herpesvirus, among others.

In the last decades, new gammaherpesviruses have been described in wild species, including felids [13], mustelids [14], other carnivores [15], and lagomorphs [16]. The order Lagomorpha comprises rabbits, cottontails, hares and pikas, which are included in two families with over 60 species. To the family Ochotonidae corresponds one single genus, *Ochotona* (pikas), while the family Leporidae comprises both rabbits and hares, including the genera *Oryctolagus* and *Lepus*. The broad distribution of lagomorphs, partly due to human introductions, spans from deserts to the Arctic, reflecting their ecological success. However, predation, unsustainable hunting practices, and habitat loss and fragmentation have led to significant declines in lagomorph populations [17, 18]. Viral diseases have also substantially contributed to this decline, particularly of the European rabbit (*Oryctolagus cuniculus*) and hare species (*Lepus granatensis*, *L. europaeus* and *L. timidus*). In fact, viruses causing high mortalities in lagomorphs, such as rabbit hemorrhagic disease virus (RHDV) and myxoma virus (MYXV), recently expanded their host range [19–24]. This added a layer of complexity to the conservation of these species due to their pivotal role in the ecosystem as prey for many endangered predators (e.g. the Spanish imperial eagle, *Aquila adalberti*, the Iberian lynx, *Lynx pardinus*, and the Iberian wolf, *Canis lupus signatus*) [25]. While the focus of the scientific community is often biased towards viruses that cause overt mortality, exposure to other apparently benign diseases that potentially affect wildlife welfare and population dynamics remains unexplored.

To date, six distinct herpesviruses have been identified in lagomorphs, with varying pathogenicity and host specificity. Leporid herpesvirus 4, LeHV-4 (species *Simplexvirus leporidalpha4*), is an alphaherpesvirus with a broad tissue tropism that is associated with severe disease and fatal infections in rabbits, producing systemic infection [26, 27]. While few cases have been reported, reinfection was observed in a farm in Alaska several months later after the first outbreak [27]. The other five herpesvirus affecting lagomorphs were tentatively classified in the subfamily *Gammaherpesvirinae*, genus *Rhadinovirus*. LeHV-2 affects the European rabbit, and is generally described as causing asymptomatic infections, but mild encephalitis and local erythema were reported in experimental infections [28]. Isolation and characterization of the virus dates back to 1969 and it was hypothesized that it corresponded to virus III described in 1923 [29, 30]. LeHV-6 also affects the European rabbit and appears to be related to concomitant infections [31, 32]. LeHV-1 and LeHV-3 were both isolated from cottontails (*Sylvilagus floridanus*) with minor differences in immunoreactivity [33]. The latter causes tumor-like lesions in several organs and is unable to establish productive infection in New Zealand white rabbits (reviewed in 27). LeHV-5 was the first herpesvirus reported in the genus *Lepus*. Iberian hares with myxomatosis presented lesions compatible with a herpesvirus infection, and a role of myxoma virus in herpesvirus infection and reactivation was hypothesized [16]. Nonetheless, apparently healthy hares were also found to be infected, with liver and spleen positive for LeHV-5 DNA, confirming the occurrence of asymptomatic infection [16].

In wildlife, the effects of herpesvirus infection have rarely been investigated, and descriptions often result from casualties or studies focusing on other pathogens. Yet, an association between gammaherpesvirus infection and reproductive fitness has been established. For instance, infection of the European badger (*Meles meles*) by mustelid gammaherpesvirus 1 affects reproductive performance [34]. Despite the potential negative impact of lagomorph herpesviruses on already declining populations, genomic data remain sparse, with only 26 sequences available in GenBank as of October 2025. Gammaherpesviruses impact both human and animal health, and are of increasing concern at the wildlife-domestic-human interface. As lagomorphs are key species in Europe [35], as well as important biological models to study human diseases and with a unique immune system [36, 37], we surveyed lagomorph species for (novel) herpesviruses. Our findings indicate widespread presence of herpesviruses in lagomorphs and uncovered a putative novel member of the family *Orthoherpesviridae*. Further studies are warranted to confirm the biological, physical and chemical properties and impact in lagomorph populations.

## Materials and methods

### Screening and PCR amplification

Lagomorph samples were collected under the scope of previous projects between 1987 and 2019; thus, all DNA material was already available at the CIBIO/InBIO, University of Porto, Portugal, facilities. In total, we tested 1,036 samples covering the study period (Supplementary Table 1). Of those, 570 were *Oryctolagus cuniculus* (466 *O. c. algirus*, 34 *O. c. cuniculus*, 69 domestic rabbits and 1 unclassified), 352 *Lepus* (168 *L. granatensis*, 117 *L. europaeus*, 41 *L. timidus*, 10 *L. castroviejoi*, 7 *L. saxatilis*, 3 *L. corsicanus*, 2 *L. capensis*, 2 *L. californicus*, 1 *L. americanus*, and 1 *L. townsendi*), 42 *Ochotona* (6 *O. hyperborea*, 5 *O. alpina*, 5 *O. dauurica*, 4 *O. hoffmanni*, 4 *O. pusilla*, 3 *O. mantchurica*, 3 *O. princeps*, 3 *O. rutila*, 3 *O. turuchanensis*, 2 *O. pallasi*, 1 *O. rufescens*, and 3 unclassified), 45 *Sylvilagus* (8 *S. bachmani*, 8 *S. floridanus*, 2 *S. cunicularius*, and 27 unclassified), 19 *Brachylagus idahoensis*, 3 *Romerolagus diazi*, 3 *Pentalagus furnessi*, and 2 *Bunolagus monticularis*. Tissues included liver, muscle, blood, skin (ear), kidney, and uterus. For the majority of cases, each sample corresponded to a single animal and tissue, except for three mountain hares (*L. timidus*), where liver and muscle were pooled in a single tube, and for ten domestic European rabbits (*O. c. cuniculus*), where liver and muscle from the same animal were tested separately. Most of the samples were from Europe: Portugal (*n* = 567), Spain (*n* = 145), Austria (*n* = 59), France (*n* = 28), Germany (*n* = 19), Sweden (*n* = 10), Italy (*n* = 10), and other European countries (*n* = 3; Ireland, Finland and Switzerland). The remaining samples were from Africa (*n* = 7), Asia (*n* = 52), and North America (*n* = 25), while one hundred and eleven samples had unknown origin.

We first screened our samples using a generalist nested PCR targeting the DNA polymerase of herpesviruses [38] using the Phusion Flash High-Fidelity PCR Master Mix (Thermo Scientific), two pmol of each primer, 0.6µL of DNA, and ultra-pure water for a final PCR volume of 10µL. The first round of amplification was carried out using forward primers DFA (5’-GAYTTYGCNAGYYTNTAYCC-3’) and ILK (5’-TCCTGGACAAGCAGCARNYSGCNMTNAA-3’) and reverse primer KG1 (5’-GTCTTGCTCACCAGNTCNACNCCYTT-3’). The second round was carried out with forward primer TGV (5-TGTAACTCGGTGTAYGGNTTYACNGGNGT-3’) and reverse primer IYG (5’-CACAGAGTCCGTRTCNCCRTADAT-3’). The expected size of secondary PCR products ranges from 215 to 315 bp [38]. On both rounds, cycling conditions consisted of 98 °C for 3 min, followed by 40 cycles of 30 s at 98 °C, 30 s at 46 °C and 30 s at 72 °C. A final extension of 5 min at 72 °C terminated the reaction. Positive results were confirmed by agarose gel electrophoresis, followed by Sanger sequencing on an automatic sequencer ABI PRISM 3500xL Genetic Analyzer (Applied Biosystems). To further validate the results, a different operator repeated the PCR for positive samples several months after the first screening.

## BLAST and phylogenetic analysis

To characterize the genetic relationships of the herpesvirus sequences obtained, Basic Local Alignment Search Tool (BLAST) analysis was performed using the NCBI Nucleotide and Protein BLAST interfaces (https://blast.ncbi.nlm.nih.gov/Blast.cgi) with the default parameters. Searches were initially optimized using Megablast to identify closely related nucleotide sequences, and when no results were obtained, BLASTn was employed to identify sequences with moderate similarity. Top hits were retrieved and aligned with our sequences, together with selected publicly available sequences of the DNA polymerase of gammaherpesvirus genera (*n* = 86). The alignment used 61 sequences produced in this study and 25 sequences from the databases. MAFFT version 7 [39] was used to generate the alignment, which was then visually inspected in BioEdit version 7.0.5.3 [40]. Pairwise genetic distances were calculated in MEGA-X [41] based on the nucleotide and amino acid alignments using the p-distance model and 1,000 replicates, with pairwise deletion of gaps/missing data. Phylogenetic trees were constructed using the Maximum Likelihood method in MEGA-X [41] with 1,000 bootstrap replicates, to evaluate the relationships between the novel herpesvirus sequences and the ones publicly available. The best-fit nucleotide and amino acid substitution models were selected by the same software. The final nucleotide alignment used for the analysis comprised 232 positions, of which 152 were phylogenetically informative. For the corresponding amino acid alignment (77 positions), 42 sites were phylogenetically informative. When considering only LeHV-5 and LeHV-6 public sequences alongside our newly obtained sequences, the number of phylogenetically informative sites was 36 for the nucleotide alignment and 10 for the amino acid alignment.

## Results

**Fig. 1:**
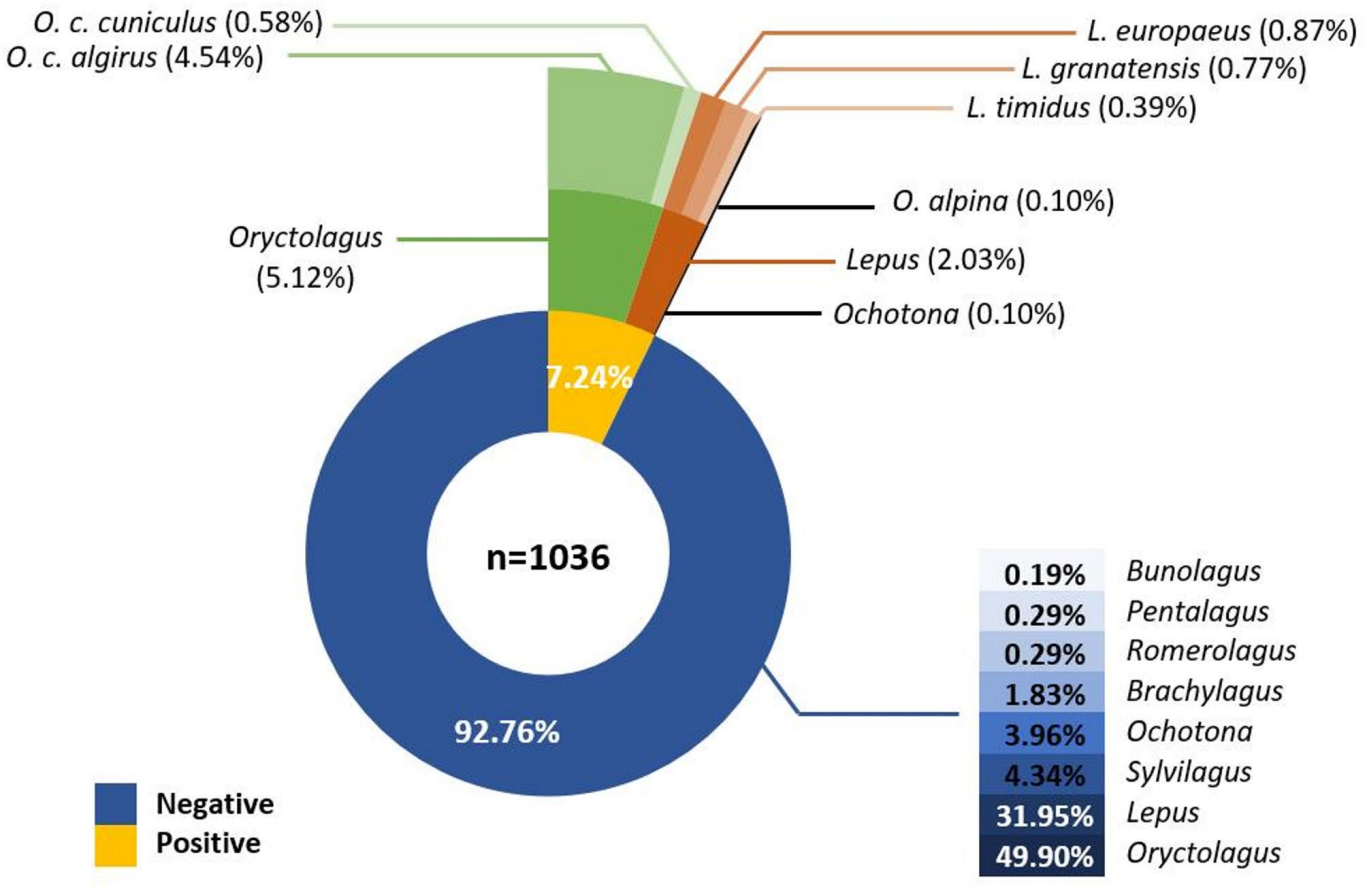
Percentage of herpesvirus-positive and -negative samples detected across different lagomorphs. Percentages are shown at the genus level. For positive samples, species-level percentages are additionally indicated. Detection was performed through PCR targeting the herpesvirus DNA polymerase with primers from [38]

Combining all sample types, we successfully amplified and sequenced herpesviruses from 75 out of the 1,036 samples analyzed (7.24%; Fig. 1). Due to the low quality and/or insufficient coverage of some sequences, we selected a total of 61 for the phylogenetic analysis, which were deposited in GenBank with accession numbers PV400185–PV400245. Of the 75 positive samples, 53 belonged to wild European rabbits (47 *O. c. algirus*, 62.67%, and six *O. c. cuniculus*, 8.00%), nine to European hares (*L. europaeus*; 12.00%), eight to Iberian hares (*L. granatensis*; 10.67%), four to mountain hares (*L. timidus*; 5.33%), and one to an Alpine pika (*O. alpina*; 1.33%). When considering the number of samples tested per species, the Alpine pika exhibited the highest prevalence (20.00%), followed by European rabbits (*O. c. cuniculus* with 17.65% and *O. c. algirus* with 10.09%). The lowest prevalence was observed in hares: *L. timidus* (9.76%), *L. europaeus* (7.69%) and *L. granatensis* (4.76%). Regarding geographical distribution, rabbit positive samples were from Portugal (including the archipelagos of Azores and Madeira), Spain and France. Positive Iberian hares were from both Portugal and Spain, positive European hares were from Germany and Austria and positive mountain hares were from France, Italy, Sweden and Switzerland, showing a widespread geographic distribution. The pika sample positive for herpesvirus was from Russia.

Notably, no herpesvirus DNA was detected in domestic rabbits, despite the number of samples analyzed (*n* = 69) that is higher than the number of wild rabbits from the same subspecies tested (*n* = 34). Likewise, viral DNA was not detected in any *Sylvilagus*, *Brachylagus*, *Romerolagus*, *Pentalagus* or *Bunolagus* species. No temporal bias is observed, since positive samples cover almost the entire study period (1988–2018). While no herpesvirus DNA was found in Africa and North America, the number of samples screened from these continents was lower (*n* = 32), representing approximately 3% of our dataset, which does not allow to exclude the possibility of viral presence in those regions. The virus was primarily detected in the liver (29 out of 75 positive samples, 38.67%), but it was also found in the skin (11/75, 14.67%), blood (6/75, 8.00%), kidney (1/75, 1.33%) and uterus (1/75, 1.33%). For 27 positive samples, the source of the DNA extraction was unknown.

BLAST analysis of our sequences (corresponding to ~ 160 bp of the DNA polymerase gene) revealed that the herpesviruses found in hares share high nucleotide and amino acid identity with leporid gammaherpesvirus 5, LeHV-5 (~ 98% and 96% with GenBank accession numbers MN557129 and QIW77237, respectively). The sequences found in rabbits share ~ 99% nucleotide identity and ~ 96% amino acid identity with LeHV-6 (GenBank accession numbers PV730058 and XUS42017, respectively), ~ 81% nucleotide and amino acid similarity with LeHV-5, followed by rodent herpesviruses with ~ 76% nucleotide and amino acid identity but lower coverage (e.g. GenBank accession numbers AYU70915 and PQ286025, respectively). The gammaherpesvirus found in pika did not retrieve any nucleotide similarity with lagomorph gammaherpesviruses, including those described in this study, but revealed ~ 86% identity with Bandicota indica rhadinovirus (GenBank accession number EF128043), although with a query coverage of only 32%. Regarding the amino acid sequence, it was mostly similar with LeHV-5 (~ 59%, GenBank accession number QJC69057). The genetic distances calculated for the sequences obtained in our study revealed that sequences retrieved from hares have an amino acid distance of 20.4% to sequences retrieved from rabbits and of 50.8% to the pika gammaherpesvirus, whilst rabbit sequences and the pika gammaherpesvirus have an amino acid distance of 53.6%. Within groups, this distance is consistently below 2%, showing a high level of conservation within host species.

**Fig. 2:**
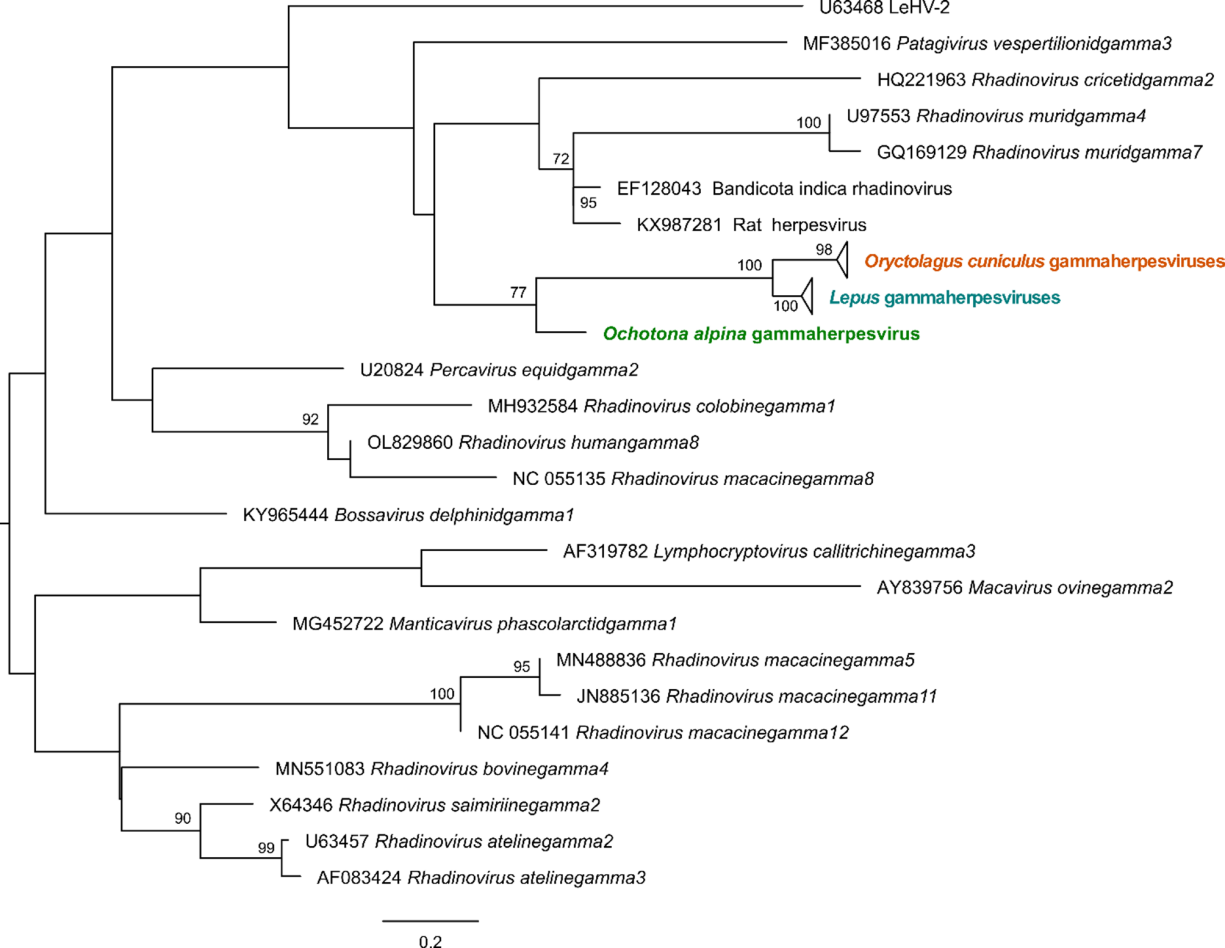
Maximum-likelihood (ML) phylogenetic tree inferred from a partial fragment of the DNA polymerase (77 aa; *n* = 86). GenBank accession numbers of sequences used are listed in the tree. Branch support was obtained from 1000 bootstrap replicates; bootstrap values are only shown for values greater than 70. Horizontal branch lengths are drawn to scale of amino acid substitutions per site. Tree was midpoint rooted. For clarity, the group of *Oryctolagus cuniculus* (in orange) and *Lepus* spp. (in blue) gammaherpesviruses were collapsed. These groups include public available sequences MN514243, MN557129, and PV730057. The sequence in green corresponds to the herpesvirus from pika

To further clarify the relationships of our sequences within the subfamily *Gammaherpesvirinae*, we performed a phylogenetic analysis with our amino acid sequences (77 amino acids) alongside representatives from each genus within the subfamily (Fig. 2). In congruence with the genetic distance analysis, our sequences cluster closely with LeHV-5. Notably, the sequence from *O. alpina* occupies a more basal position within this group (bootstrap value of 77), supporting an earlier evolutionary divergence of pika herpesviruses compared to those found in hares and rabbits. The rabbit (*O. cuniculus*) and hare (*Lepus* spp.) herpesviruses form distinct clusters (bootstrap values > 98), further supporting their uniqueness within the subfamily. The phylogenetic tree also confirms that our sequences are not closely related to LeHV-2.

## Discussion

Herpesviruses are thought to have co-evolved with their hosts over extensive periods of time, resulting in exquisite well adaptation and exceptionally restricted host tropism. Recent discoveries of novel herpesviruses in wild populations [16, 31, 42, 43] suggest that lagomorphs may also follow a pattern of species-specific herpesvirus-host associations. However, knowledge about their presence and prevalence in both wild and domestic lagomorphs remains scarce. Here, we present the findings from the first comprehensive screening of the presence of herpesviruses in lagomorph species. Our study detected viral sequences in multiple individuals, across different host species and sampling periods. To validate these results, PCR-positive samples were independently retested by different operators several months apart, reducing the likelihood of laboratory artifacts. Additionally, we analyzed high-throughput sequencing (HTS) data of rabbit and hare genomes (data not shown) to rule out the possibility that these sequences represent integrated viral fossils rather than biologically active viruses. No gammaherpesvirus sequences were recovered from host genomic data, confirming the validity of our results.

Herpesvirus prevalence among wild populations can be quite variable, from 9.9% in free-ranging cervids to 51% in northern brown bandicoots, and 64% in wild bobcats, for example [42–44]. Regarding lagomorph herpesviruses, a previous study found a prevalence of 41.17% in wild rabbits, despite the low number of samples analyzed (*n* = 34) [32]. In our study, prevalence appears to be lower (7.24%; Fig. 1); however, viral detection may be underestimated for several reasons. For instance, the type of tissue significantly impacts detection, due to the presence of variable viral loads across different tissues. Indeed, in petaurid possums, spleen performed better than liver or lung [45], while in black bears (*Ursus americanus*), the spleen and lymph nodes tested positive, but not liver or kidney [46]. Here, no spleen samples were screened, thus no inference can be taken regarding herpesvirus presence in spleen compared to other tissues. Additional factors, such as the time of sampling, geographic location, and assay sensitivity, can also contribute to detection biases [47]. Furthermore, during latency, viral loads may be too low for PCR detection, making it challenging to accurately estimate the true prevalence of this virus. These limitations should be accounted for in future studies to refine our understanding of herpesvirus distribution, especially in light of potential spillovers from domestic animals to wild populations and vice-versa.

An underestimation of infection prevalence, influenced by the phase of viral infection (lytic vs. latent), can have significant implications when trying to link infection by gammaherpesvirus with other pathological conditions, and in defining their consequence for diagnostic and therapeutic intervention. For example, individuals infected with equine herpesvirus 2 are more prone to secondary infections [48]. Equine herpesvirus 2 appears to be related to the development of pneumonia, with vaccinated horses being less susceptible to the disease [48]. Particularly relevant for our study is the co-infection of MYXV and/or RHDV with herpesviruses in rabbits [31] and hares [16]. LeHV-5 was identified in hares co-infected with MYXV that presented clinical signs not compatible with a poxvirus infection [16]. MYXV-induced immunosuppression may facilitate herpesvirus replication and/or reactivation, as the transition from latency to lytic infection is often triggered by stress, disease, or similar factors. In Europe, the widespread practice of translocating or rearing animals in captivity for hunting management, namely wild rabbits and European hares, introduces additional stress, which could drive viral reactivation. In any case, as asymptomatic/healthy individuals with no signs of disease can shed and transmit the virus to vulnerable hosts (e.g. 49), acting as a source of viral transmission, gammaherpesvirus infections remain a challenge to the management of wild species.

The DNA polymerase, a key enzyme in the lytic phase of infection that ensures the replication of the viral genome, is among the most conserved genes in herpesviruses [50], yet contains sufficient sequence variation to differentiate between species, making it an ideal candidate for initial viral identification [38]. Our analysis revealed three distinct clusters: one grouping all rabbit sequences, another containing hare sequences, and a third comprising the pika herpesvirus (Fig. 2). This pattern shows a high level of conservation among closely related species, while sequences between groups show significant divergence, and indicates that the European rabbit (*O. cuniculus*), for example, harbors a genetically distinct gammaherpesvirus compared to hares. The presence of separate clades for different lagomorph herpesvirus supports that host-virus co-evolution has played a role in herpesvirus diversification [51]. The clustering of lagomorph herpesviruses with rodent gammaherpesviruses also suggests a possible evolutionary link between herpesviruses of the superorder Glires. This supports the hypothesis that gammaherpesviruses evolved through multiple interspecies transmission events, followed by host-specific adaptation and divergence [52]. Notably, in the Blast analysis, the pika herpesvirus exhibited amino acid sequence similarity with LeHV-5 but lacked nucleotide similarity, suggesting that most substitutions are silent and compatible with the pivotal role of the encoded protein.

Altogether, our findings suggest that the herpesviruses sequences identified in rabbits in this study may represent divergent gammaherpesviruses within this family, which was tentatively named as LeHV-6 by other authors [32]. Similarly, the gammaherpesvirus detected in pika appears to represent a distinct genus based on genetic distance and phylogenetic placement. While its divergence suggests the possibility of a new genus, further genomic and biological data will be required before a formal classification can be proposed. In *Oryctolagus*, a gammaherpesvirus (LeHV-2) has previously been described [28]; however, our analysis reveals significant differences between LeHV-2 and the sequences identified in our study, which are more closely related to LeHV-5 (Fig. 2). For LeHV-1 and LeHV-3, associated with *Sylvilagus* infection [33], no genetic analyses are available. While it cannot be ruled out that LeHV-6 or the pika gammaherpesvirus may correspond to these gammaherpesviruses previously described in cottontails, this seems unlikely given the strong host-specificity of gammaherpesviruses [53]. Indeed, our sequences originate from distinct host species and exhibit high divergence compared to known herpesviruses. For context, closely related members of the genus *Rhadinovirus* show lower amino acid divergence in the same genomic region (e.g. *Rhadinovirus atelinegamma2* vs. *Rhadinovirus atelinegamma3*, 4.3%; *Rhadinovirus muridgamma4* vs. *Rhadinovirus muridgamma7*, 6.6%; *Rhadinovirus macacinegamma5* vs. *Rhadinovirus macacinegamma12*, 13.7%;).

Considering the ecological and biological significance of lagomorphs, it is crucial to understand the impact of gammaherpesvirus infections on disease ecology and lagomorphs’ health, as their importance extends far beyond wildlife and production systems. A comprehensive understanding of these viruses requires not only broad surveys of virus occurrence in both wild and domestic animals, but also in-depth research into their association with inexplicable mortality or atypical/non-related health conditions. Fertility-related issues, such as abortion and other reproductive disorders, are important drivers of population dynamics, and may be related to apparent benign herpesvirus infections without overt clinical manifestations [54], putting wild populations at risk. Another critical factor to consider is the role of sympatric species in facilitating cross-species transmission, as gammaherpesviruses can remain benign in their reservoir hosts but cause disease in other species (e.g. [55]), leading to fatal lymphoproliferative disease. Gammaherpesviruses can also act as significant co-factors in other diseases, such as HIV and malaria [56]. Additionally, given their ability to modulate the host immune response [56], studying these apparently benign infections is crucial, especially since immunocompromised individuals are more likely to develop disease, whereas healthy individuals typically remain asymptomatic [57].

## Conclusions

In conclusion, to our knowledge, this is the first report of gammaherpesviruses in pikas (*Ochotona alpina*), European hares (*Lepus europaeus*), and mountain hares (*Lepus timidus*), along with a genetic characterization that documents their widespread occurrence across Europe. These results are intended to facilitate discussion and promote further investigation, rather than being formal taxonomic assignments. While the genetic data obtained in this study are limited to a single gene, they provide the foundation for genetic assignment and underscore the need for broader genomic analyses to provide a definitive classification and unravel their evolutionary relationships within the *Herpesviridae* family. Further studies to clarify the clinical significance of these infections and how this may affect wildlife and production systems are warranted.

## Supplementary Information

Supplementary Material 1.

## Data Availability

The data that support the findings of this study are openly available in GenBank at https://www.ncbi.nlm.nih.gov/, with accession numbers PV400185–PV400245. The GenBank accession numbers of sequences used in the phylogenetic analysis are listed in Fig. 2.
